# Visualizing Adsorption of Cyanophage P-SSP7 onto Marine *Prochlorococcus*

**DOI:** 10.1038/srep44176

**Published:** 2017-03-10

**Authors:** Kazuyoshi Murata, Qinfen Zhang, Jesús Gerardo Galaz-Montoya, Caroline Fu, Maureen L. Coleman, Marcia S. Osburne, Michael F. Schmid, Matthew B. Sullivan, Sallie W. Chisholm, Wah Chiu

**Affiliations:** 1 National Center for Macromolecular Imaging, Verna and Marrs McLean Dept. of Biochemistry & Molecular Biology, Baylor College of Medicine, Houston, TX, 77030, USA; 2 National Institute for Physiological Sciences, 38 Nishigonaka, Myodaiji, Okazaki, Aichi, 444-8585, Japan; 3 School of Life Sciences, State Key Lab for Biocontrol, Sun Yat-Sen University, Guangzhou, 510275, P. R. China; 4 Department of Civil and Environmental Engineering, M.I.T., Cambridge, MA, 02139, USA.

## Abstract

Marine cyanobacteria perform roughly a quarter of global carbon fixation, and cyanophages that infect them liberate some of this carbon during infection and cell lysis. Studies of the cyanobacterium *Prochlorococcus* MED4 and its associated cyanophage P-SSP7 have revealed complex gene expression dynamics once infection has begun, but the initial cyanophage-host interactions remain poorly understood. Here, we used single particle cryo-electron tomography (cryo-ET) to investigate cyanophage-host interactions in this model system, based on 170 cyanophage-to-host adsorption events. Subtomogram classification and averaging revealed three main conformations characterized by different angles between the phage tail and the cell surface. Namely, phage tails were (i) parallel to, (ii) ~45 degrees to, or (iii) perpendicular to the cell surface. Furthermore, different conformations of phage tail fibers correlated with the aforementioned orientations of the tails. We also observed density beyond the tail tip in vertically-oriented phages that had penetrated the cell wall, capturing the final stage of adsorption. Together, our data provide a quantitative characterization of the orientation of phages as they adsorb onto cells, and suggest that cyanophages that abut their cellular targets are only transiently in the “perpendicular” orientation required for successful infection.

Microbes drive the nutrient and energy transformations that sustain Earth’s ecosystems^1^, and the viruses that infect them modulate both microbial population size and diversity^2^,^3^,^4^,^5^,^6^. The cyanobacterium *Prochlorococcus,* the most abundant oxygenic phototroph on Earth, contributes a substantial fraction of global primary carbon production, and often reaches densities of over 100,000 cells per milliliter in oligotrophic and temperate oceans^7^,^8^,^9^. Hence, viral (cyanophage) infection and lysis of *Prochlorococcus* represent an important component of the global carbon cycle. In addition to their ecological role in inducing host mortality, cyanophages influence the metabolism and evolution of their hosts by co-opting and exchanging genes, including core photosynthesis genes^10^,^11^,^12^. Cyanophage P-SSP7, the focus of this study, has become a model system for exploring these interactions. Its genome shares similarities with T7-like podovirus genomes, but is augmented with several metabolic genes that appear to be derived from its host^13^,^14^. Cyanophage-encoded photosynthesis genes are transcribed and translated during infection of its host, and this has been proposed to help maintain photosynthesis in infected cells^15^,^16^. These auxiliary metabolic genes, or AMGs^17^, are widespread among cyanophages in isolate cultures^11^,^12^,^13^,^14^,^18^,^19^, and in the wild^20^,^21^,^22^.

Cyanophage P-SSP7, which has podovirus morphology^23^, has been studied at a structural level using both cryo-electron microscopy (cryo-EM) and cryo-electron tomography (cryo-ET)^24^. Such studies revealed that it has a linear double-stranded DNA genome packed into a ~655 Å diameter non-enveloped capsid arranged in a T = 7 icosahedral lattice. One of the twelve capsid vertices harbors a short (about 230 Å in length), non-contractile tail hub that is surrounded by six thin tail fibers. The capsid protein of P-SSP7 is structurally similar to that of other phages, including podoviruses [T7^25^, ε15^26^,^27^, P22^28^, HK97^29^ and Syn5^30^] and myoviruses [T4^29^] – all of which have the same protein fold^27^,^31^. In contrast, the structures of the tail hub and tail fibers are quite different among these phages, likely reflecting strong evolutionary pressure to optimize interactions with their hosts. In *Salmonella* phages P22 and ε15, for example, six trimeric tailspikes (which differ from tail fibers in that they are more rigid) are bound in grooves of the tail hub; each has head binding, hinge and receptor binding domains, and the tips of the tailspikes point away from the capsid^32^. In phages T7 and P-SSP7, the tail fibers are highly flexible; however, their tips appear to bind to the capsid instead of pointing away from it^33^. These differences among phages suggest that their corresponding adsorption mechanisms onto their hosts may also differ. It has been suggested that tail fiber orientation may affect not only host recognition, but also the interactions between the tail fiber, the adaptor, and the portal, which are necessary to trigger internal core protein disassembly and DNA release^24^. Cryo-ET and subtomogram averaging have been increasingly used to study the interactions between phages and host cells *in situ*; for example, for *Salmonella* podovirus ε15^34^, *Escherichia coli* podovirus T7^33^, and *Synechococcus* podovirus Syn5^35^. For ε15, no structural rearrangement of the tail fibers was seen upon cell surface attachment, nor did the phages appear to change their orientation with respect to the cell membrane. In contrast, phage T7 tail fibers adopt various conformations during adsorption. Although the adsorption structure of biochemically purified Syn5 has been reported, a systematic study on whether its tail fibers change in conformation during phage adsorption onto the host bacterium is lacking. For *Prochlorococcus* podovirus P-SSP7, the focus of this study, our previous single particle cryo-EM study comparing DNA-containing (full) and DNA-released (empty) phages revealed extensive tail fiber rearrangements between these two states^24^. This suggests that infection of host cells by P-SSP7 might require such rearrangements. Here, we used cryo-ET to quantitatively characterize phage orientations with respect to the cell membrane and tail fiber conformations on a per-particle basis. Indeed, we observed tail fiber rearrangements *in situ* during P-SSP7 adsorption onto cyanobacteria.

## Results and Discussion

### Experimental overview and initial screenings

Cryo-ET was applied to samples collected from time-course experiments designed to capture different stages in the adsorption cycle of P-SSP7 onto *Prochlorococcus*. Briefly, *Prochlorococcus* cells in late exponential phase were concentrated to ~10^9^ cells/ml by centrifugation and mixed with ~40-fold the number of phages. The samples were imaged from P-SSP7 adsorption through cell lysis, up to ~386 min post-infection (m.p.i., see Methods).

**Figure 1:**
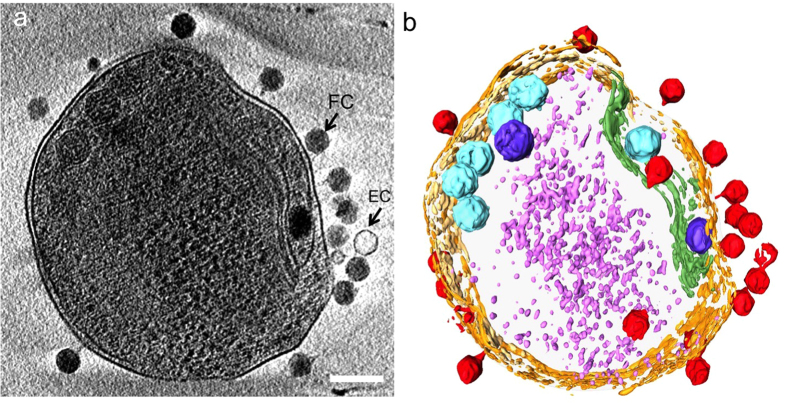
Adsorption of P-SSP7 phage to *Prochlorococcus* MED4 visualized by cryo-ET. (**a**) Slice (~20 nm) through a reconstructed tomogram of P-SSP7 phage incubated with MED4, imaged at ~86 min post-infection, and (**b**) corresponding annotation highlighting the cell wall in orange, the plasma membrane in light yellow, the thylakoid membrane in green, carboxysomes in cyan, the polyphosphate body in blue, adsorbed phages on the sides or top of the cell in red, and cytoplasmic granules (probably mostly ribosomes) in light purple. FC and EC show full-DNA capsid phage and empty capsid phage, respectively. Scale bar is 200 nm.

**Figure 2:**
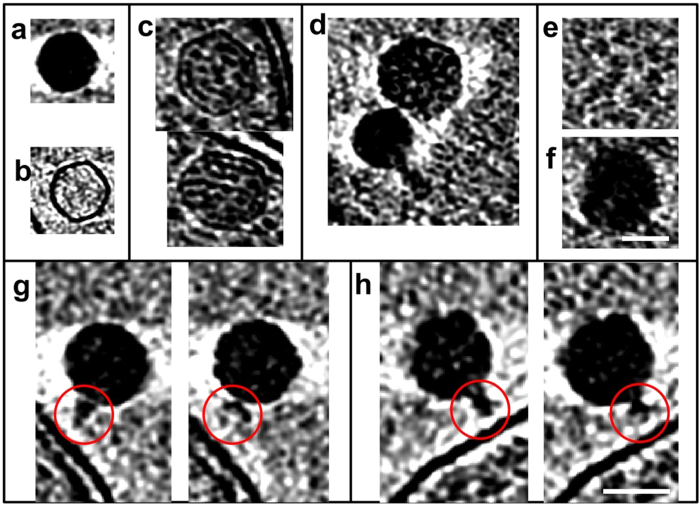
Subcellular features seen in cryo-ET of *Prochlorococcus* MED4 cells infected with P-SSP7 phages. P-SSP7 capsids (**a**) full of DNA, and (**b**) empty. (**c**) Carboxysomes inside MED4 cells. (**d**) P-SSP7 capsid (lower) and carboxysome (upper). (**e**) Ribosome-like particles. (**f**) Polyphosphate body. (**g**) Two views of the same P-SSP7 virus at different z-slices in a tomogram, showing tail hub and tail fiber densities (red circles) changing with Z-slice. (**h**) A second example similar to (**g**). The displays of (g) and (h) are made to optimize the visibility of the tail and tail fibers. The scale bar is 50 nm.

**Figure 3:**
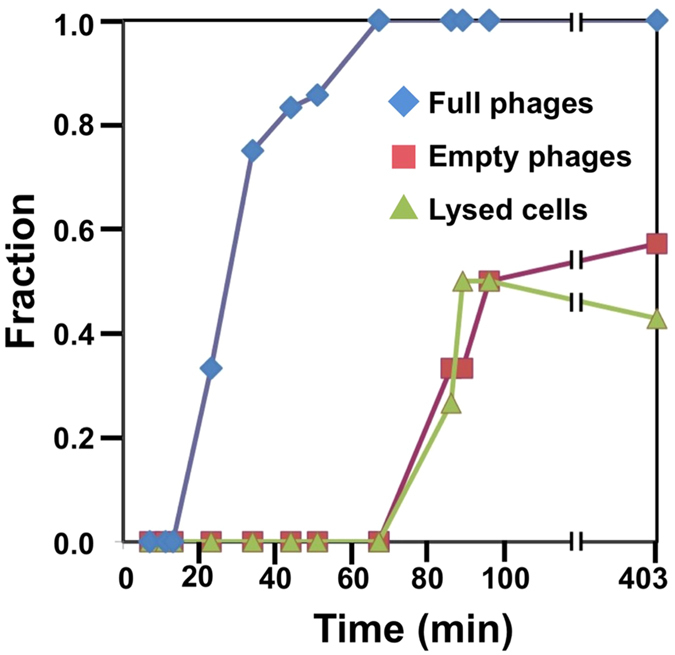
Time course analysis of P-SSP7 phage infection of *Prochlorococcus* MED4 cells. Pre-infection phages (capsids filled with DNA or “full”, blue line) began to appear close to (*i.e.* less than the diameter of the capsid) the cells at ~23 min after initiation of infection. Post-infection phages without DNA (“empty”, red line) were first observed at ~86 min after initiation of infection. Lysed cells (green line) were also first detected at ~86 min after initiation of infection. The vertical axis shows the fraction of the tomograms exhibiting each of the features at each time point.

Preliminarily screening of ~90+ grids frozen at different post-infection time points revealed almost no intact cells after 7 h post-infection. Grids that were significantly bent, devoid of sample, or that yielded thick ice were discarded. As a result, 64 grids were suitable for data analysis (Supplementary Table). Among these, the 16 best ones were selected for tiltseries collection based on ice thickness, phage-cell concentration, and infection time diversity. In the end, 70 suitable tiltseries were reconstructed into tomograms, corresponding to time points between 11 and 386 m.p.i. Many biological features are visible in our tomograms, as shown in Fig. 1a and annotated with different colors in Fig. 1b (also, see Movies S1, S2, S3). Prominent features include phages full of DNA (Fig. 2a,d,g,h), empty phages (Fig. 2b), and cellular features such as the cell wall and plasma membrane, thylakoid, carboxysomes (Fig. 2c,d), ribosome-like particles (Fig. 2e), and dense granules (Fig. 2f). Different numbers of phages were adsorbed onto the cell surface depending on post-infection time (Supplementary Fig. 1). An analysis of our time-course of tomograms (Fig. 3) showed that phage particles were not detectably attached to the cell surface until ~23 m.p.i. Furthermore, phages did not appear to release their genome into their host until ~86 m.p.i., when empty capsids (*i.e.*, phages devoid of DNA) were first observed. Of note, cells began to lyse also at ~86 m.p.i. Our imaging-based observations are consistent with a prior characterization of this system using PCR-based approaches^16^, except that we observed lysis at even earlier infection time points.

### Classifying and quantifying cyanophage particle orientations during adsorption

**Figure 4:**
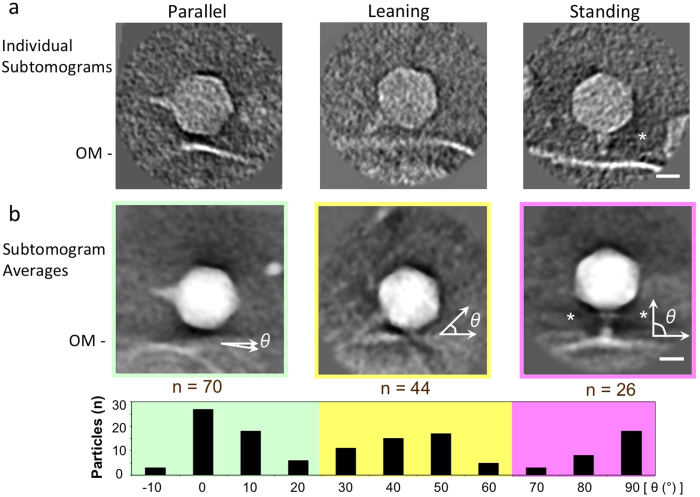
Adsorption processes of P-SSP7 phages onto *Prochlorococcus* MED4. Subtomograms of full phages close to cells (n = 140; *i.e.*, excluding “free” full-capsid phages far away from cells) were classified into three groups based on the tail orientation with respect to the cell surface: “parallel”, “leaning” and “standing”. (**a**) Representative images of a single phage in each class. (**b**) Top panel: subtomogram averages for each class. Lower panel: Number of phage particles with a particular angle of the tail with respect to the cell surface, with vertical (perpendicular) orientation considered as 90°. 70 phages (50%) are classified as “parallel” (left), in which the tail hubs are almost parallel to the cell surface (θ ≈ 0°) and do not appear to directly interact with the cell wall (outlined in green). 44 phages (31%) are classified as “leaning” (middle), in which the tail is at an angle to the cell surface (θ ≈ 45°) and partly interacts with it (outlined in yellow). 26 phages (19%) are classified as “standing” (right), in which the tail is vertically connected to (θ ≈ 90°) and completely interacts with the cell surface (outlined in purple). * Horizontally extended tail fibers are visible in the subtomogram average of “standing” phages. OM denotes the outer membrane of the host cell. Scale bar is 20 nm.

**Figure 5:**
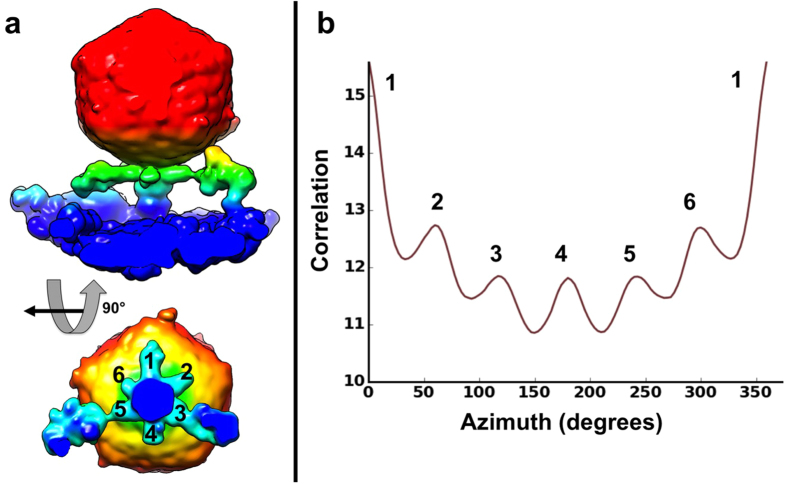
Tail of P-SSP7 phage adsorbed onto *Prochlorococcus* MED4 cell shows pseudo six-fold symmetry. (**a**) Isosurface views of a P-SSP7 average (“standing” class in Fig. 4) without any imposed symmetry, showing tail and tail fiber densities in green hues (the main capsid body is in red hues). (**b**) Self-rotational correlation plot, demonstrating the presence of strong pseudo-six fold symmetry in the tail of the P-SSP7 average shown in (**a**). The cell surface is colored in blue.

To better understand the interactions between MED4 and P-SSP7, we further examined our 16 reconstructed tomograms. Briefly, 178 manually selected subtomograms of “full” P-SSP7 particles close to the cell were computationally extracted (see Methods). We classified them according to the angles of their tails with respect to the cell surface (Fig. 4a), and then computationally aligned and averaged the particles within each class (see Methods). The angle of interaction between phage and host cell was not uniform across particles. Instead, three main orientations were observed, corresponding to angles of ~0° (“parallel”; n = 70), ~45° (“leaning”; n = 44) and ~90° (“standing”; n = 26) (Fig. 4b) between the phage tail and the cell surface. The remaining full capsids (n = 30) were not close enough to interact with the cells, and were designated as “free”. Of note, eight subtomograms were not included in the analysis due to orientation ambiguity. If the distribution of phage orientations is reflective of their prevalence in natural systems, we might infer the following: First, phages on the host surface appear to spend considerable time with their tail hubs parallel to the cell surface, perhaps “exploring” the cell surface using tail fibers to identify cellular receptors. For T4-like phages, this is a well-known phenomenon mediated by the reversible binding of long tail fibers and the irreversible binding of short tail fibers^36^. For T7-like phages, however, phage tails and tail fibers are less prominent^37^. The idea that phages “walk” along the cell surface has also been suggested for phage T7^38^, but has not been quantitatively demonstrated. Second, of the three major orientations observed, only the “standing” class represents an adsorbed phage poised for successful DNA injection, and this class comprised only 19% of observed full-capsid phages. This suggests that either few cyanophage-host contacts result in successful adsorption and genome injection, or that “infection competent” conformations (*i.e.*, “standing” particles) are short-lived. Interestingly, tail fibers might interact asymmetrically with the cell surface during adsorption, as seen for T7^33^. For example, although we resolve clear density to account for all fibers at the base of the tail without imposing symmetry (Fig. 5a, Supplementary Fig. 2), the density of some fibers is stronger and resolved over a longer range than that of others. Indeed, strong pseudo-six-fold symmetry is evident in self-correlation plots (Fig. 5b, see Methods).

### Classifying and quantifying cyanophage tail fiber orientations during adsorption

**Figure 6:**
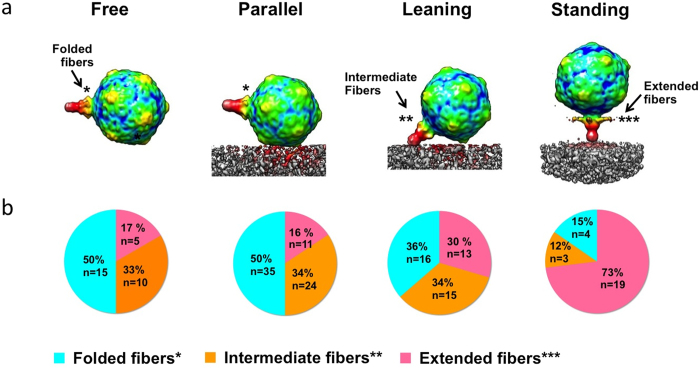
Structural change of the tail fibers correlated with stages of adsorption. (**a**) P-SSP7 averages comprised of particles classified by tail fiber conformation (***extended, **intermediate, and *folded), rotated to the orientation angle with respect to the cell surface in which each corresponding class is most prevalent. Six- and five-fold rotational symmetry was applied to the tails and capsids, respectively, to enhance structural features. (**b**) The fraction of particles with “extended”, “intermediate”, and “folded” fibers during phage adsorption in each orientation class (free, parallel, leaning, standing).

Previous cryo-EM studies showed that tail fiber conformation differs between biochemically purified full and empty P-SSP7 phage particles, suggesting that it might serve a specific function during adsorption^24^. Here, we examined the tails of 170 full phages by extracting them from phage subtomograms and classifying them according to their characteristic Eigen images (Supplementary Fig. 3a; see Methods). We discovered three major groups (Fig. 6, upplementary Fig. 3b): (i) “folded fibers” (n = 70, where fibers near the tail hub ran along the capsid surface and the tail hub showed a smooth cone shape; (ii) “extended fibers” (n = 48), where the proximal section of the fibers extended horizontally, the tail hub was slightly shorter than that with folded fibers, and the tip of the tail hub showed a round shape; and (iii) “intermediate fibers” (n = 52), likely encompassing an average of folded and extended fibers within individual phages. Taken together, tail orientation and phage adsorption data suggest that tail fiber conformation is likely to have a biological function. Specifically, for free phages that are proximal to but not interacting with the cell surface, only 17% of tail fibers were in the extended position. Similarly, only 16% of the “parallel’’ phages showed extended fibers. In contrast, 30% of the “leaning’’ and 73% of the “standing’’ phages showed tail fibers in the extended conformation (Fig. 6b). Thus, a majority of “parallel’’ and “leaning’’ phages exhibited folded fibers, while the majority of “standing’’ phages had extended fibers. The fiber-base conformations we found here are consistent with those found in our previous single particle analysis study^24^. Namely, the basal region of tail fibers was in a “folded” conformation in DNA-full phages. In our P-SSP7 data here the full length of the fibers is not visible because of their structural flexibility and the limited resolution of cryo-ET. Nonetheless we successfully resolved conformational differences at the fiber-base regions (*i.e.*, where fibers are anchored to the tail hub) of phages at different stages of adsorption.

Our data, particularly our “standing” phage average where extended fibers are seen interacting with the cell surface, suggest that tail fibers are essential to phage adsorption onto their host. Indeed, temperature-sensitive mutants of gp17 in bacteriophage T3 yielded fiberless, non-infectious phages at high temperature^39^. Similarly, in bacteriophage T7, a gp7.3 mutant with compromised tail fibers yielded full particles that failed to adsorb onto host cells^40^. A fiberless P-SSP7 mutant has not been studied; however, tail fiber conformations seem to exert the same function in adsorption as those in other bacteriophages.

Our findings suggest a role for tail fiber extension in aiding the adsorption process. On the other hand, in the absence of the host, folded tail fibers are likely adhered to the phages for protection. Tail fiber conformational changes have also been observed for phage T7, where the number of tail fibers bound to the host cell surface gradually increases during adsorption^33^, but not for phage ε15^34^. Here, our results suggest that conformational changes do occur in P-SSP7 tail fibers during adsorption.

### Formation of tail extension for DNA injection

**Figure 7:**
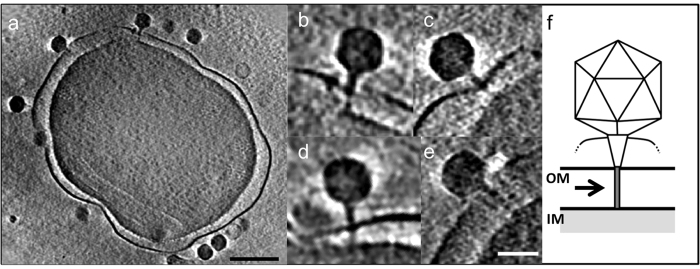
Structure of the tail extension. (**a**) Slice (~20 nm) through a reconstructed tomogram of P-SSP7 phage incubated with MED4 [scale bar is 200 nm]. (**b–e**) Multiple phages at different z-slices of (**a**) showing an extensible tube going through the cell surface, ~500 Å or longer, and half the width of the tail [scale bar is 50 nm]. (**f**) Schematic drawing of the extensible tube labeled with dark gray.

Although cyanophage P-SSP7 is a T7-like phage that lacks a contractile sheath (as is the case for T4-like phages), comparative genomics suggests that the P-SSP7 genome encodes homologs to some T7 phage tail proteins. By analogy to T7, it was hypothesized that the P-SSP7 tail releases core proteins (gp14/15/16) just prior to DNA injection to digest the bacterial envelope and build a long, narrow, extensible channel from the tip of the tail to protect viral DNA during transport into the cell^13^,^16^. Our cryo-ET observations here are consistent with these genomics-inferred hypotheses. For example, in plasmolysed areas, we observed a tail tube extending from several standing phages, ~500 Å or longer and half the width of the tail, which pierced through the cell wall and cell membrane (Fig. 7). Such tubular features were also observed for ε15, T7 and syn5 phages during infection of *Salmonella, Escherichia coli* and *Synechococcus* cells, respectively^33^,^34^,^35^. These observations are consistent with the hypothesis, derived from comparative genomics, that P-SSP7 has an extensible tail homologous to that of T7^33^.

### Towards a mechanistic model of cyanophage adsorption

**Figure 8:**
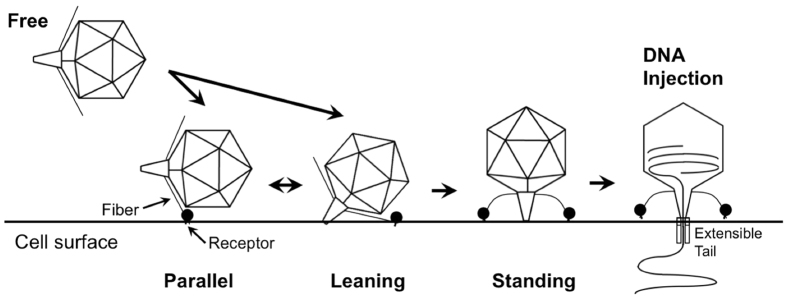
Schematic summary of the process of P-SSP7 phage adsorption onto *Prochlorococcus* MED4. The phage first adheres to the cell surface with its tail parallel to (Parallel) or at an angle (Leaning) to the cell surface in the pre-infection stage. The tail then firmly attaches to the cell surface and extends its fibers horizontally (Standing), rendering the phage infection-competent, after which viral DNA is released into the cell through an extensible tube.

Based on our per-particle observations, we propose the following conceptual model to describe the adsorption process of phage P-SSP7 onto host *Prochlorococcus* MED4 (Fig. 8): Free phages, which have not yet interacted with the bacterial cell wall, approach the cell with folded fibers and then adhere to the host cell surface with the tail hub almost parallel or at an angle to the cell surface: the “parallel” and “leaning” states being indicative of pre-infection. Once adhered, and after a presumed “walking” stage where tail fibers search for receptors on the cell surface, some tail fibers appear to attach more firmly and to extend horizontally, enabling the phage to “stand”. These conformational changes in the tail fibers might then trigger changes in the tail-portal structure, allowing the tail to firmly attach to the cell surface and release internal phage proteins to the cell membrane. Finally, this fully infection-competent “standing” state leads to the release of viral DNA into the cell. This multi-stage process may serve to ensure that the phage is in the proper orientation to productively deliver its DNA into the host. Furthermore, phages might be able to use their tail fibers to assess whether or not a cell is able to host a productive infection. Similar hypotheses have been suggested for soil mycophages (*e.g.*, “waking proteins”)^41^, and uncultured phages from the oceans^42^. Conventional (plastic-embedded, stained and/or sectioned) observations using electron microscopy showed that all the classic T1 to T7 phages adsorb onto 200 to 400 specific positions (adhesion zones) on the cell surface, presumably where the wall and protoplasmic membrane adhere to each other^43^. In contrast, we observed only a few cases per cell where an infecting phage localized to regions where the space between the membrane and the wall was narrowed (Supplementary Fig. 4). Furthermore, no such adhesion zones were observed with phage T7 infecting *E. coli* when samples were prepared using the same cryo-ET protocol^33^. Together, these observations suggest that abundant “adhesion zones” in previous studies using conventional electron microscopy might correspond to artifacts stemming from chemical fixation.

## Conclusions

Our cryo-ET experiments, including subtomogram classification and averaging, provide a first look *in situ* at the direct interactions between P-SSP7 and the marine cyanophage *Prochlorococcus* MED4. Our observations suggest that productive genome injection after the adsorption of P-SSP7 onto MED4 is either rare or transient. Furthermore, adsorption itself is shown to be a multi-stage process. Lastly, our data are consistent with the genomics-based hypothesis of the formation of an extensible tube preceding infection. While cryo-ET is the best method to visualize interactions between macromolecules *in situ*^44^, it remains challenging to establish a quantitative understanding of such nano-scale events given the relatively low-throughput of this imaging modality. Nevertheless, our analyses of 170 near-cell phage particles paint a picture of phage-host interactions that will help guide future experiments across diverse phage-host model systems.

## Methods

### Preparation of P-SSP7 phage

Phages were prepared as previously described^13^. P-SSP7 particles were propagated in *Prochlorococcus* MED4. The particles were then precipitated with polyethylene glycol 8000, purified on a cesium chloride step gradient (steps were ρ = 1.30, 1.40, 1.50, and 1.65), spun at 104,000× g for 2 h at 4 °C, and dialyzed against a buffer containing 100 mM Tris-HCl (pH 7.5), 100 mM MgSO^4^, and 30 mM NaCl. Just before use, the phages were ultracentrifuged at 104,000x g for 1.5 h at 4 °C, and the pellets were re-suspended in buffer to yield a concentration of about 10^12^ particles/ml.

### Infection of MED4 cells with P-SSP7 phages

*Prochlorococcus* MED4 host cells were grown in a modified Percival incubator on a light-dark cycle with simulated sunrise and sunset (0–110 μmol photons m^−2^ sec^−1^ light that ramps up or down for 5 h at sunrise/sunset with 4 h of stable light in the middle and 10 h of darkness) at a temperature of 22–24 °C depending on the time of day. This yielded a growth rate of approximately 0.65 day^−1^. In late exponential phase, these cells were concentrated by centrifugation to ~10^9^ cells/ml. For infection, 100 μl of cells (~10^9^ cells/ml) were mixed with 400 μl of phage (~10^10^ phage/ml), to achieve a multiplicity of infection (MOI) of ~40 virus particles per cell. Then, 5 μl aliquots of the cell-phage mixture were taken at 2–3 min intervals from 4 to 128 min, and from 381 to 403 min post infection (see Supplementary Table). All aliquots were mixed with 1 μl of 150 Å colloidal gold (Catalog No. 25–40, Electron Microscopy Sciences), applied to previously glow-discharged R3.5/1 copper Quantifoil grids (Quantifoil Micro Tools GmbH), and rapidly frozen in liquid ethane using a Leica plunger (EM-CPC, Leica Microsystems) after blotting with Whatman No. 1 filter paper. Grids of cell-phage mixture were frozen and stored in liquid nitrogen.

### Cryo-electron tomography

Imaging was done using a JEM3200FSC electron microscope (JEOL Inc., Japan) equipped with a field emission electron source operated at 300 kV and an in-column energy filter (slit width: 20 eV). Condenser and objective apertures were set at 50 and 60 μm respectively. The specimens were kept at −170 °C with liquid nitrogen. Tilt series images were collected using SerialEM automated data acquisition software^45^ in a range of ±62° at 2° increments. The images were recorded on a 4k × 4k pixels slow scan CCD camera (Gatan Inc.) at a nominal magnification of × 20 K, which resulted in an imaging resolution of ~5.4 Å/pixel. The total electron dose on the specimen per tilt series was kept under 80 electrons/Å^2^ to minimize radiation damage. The tilt series were aligned using gold fiducials, and tomograms were reconstructed using filtered back projection or SIRT in IMOD, as previously described^46^.

### Subtomogram classification and averaging

In this phage adsorption study, 178 subtomograms of infecting phages attached to or near the cell surface were extracted from 16 tomograms of phage-infected cells. Subsequent post-tomographic image processing was performed using EMAN^47^ with the following steps. First, subtomograms subjected to a 20 Å low-pass filter were aligned to the single-particle icosahedral 3D map of P-SSP7 (EMDB 1713), where a tight spherical mask was used to exclude the tail. Second, these subtomograms were further aligned with an appropriate mask including the tail, and the tails were manually oriented to all point in the same direction in Z. Aligned particles were classified according to the angle the tail made with the host cell surface, and averaged in Fourier space, weighted to reduce distortions caused by the missing wedge^48^. Eight subtomograms yielding ambiguous orientations were discarded. We also performed self-symmetry analysis^49^ on our subtomogram averages and multivariate statistical analysis (MSA) of the tail structures using EMAN2 software^50^ to classify the fiber structures surrounding the tail hub in each adsorption class. Tail subvolumes were extracted from full phage subtomograms, 6-fold symmetry was enforced, and tails were classified into three groups by MSA (Supplementary Fig. 3) to evaluate the conformation of the basal part of the fibers relative to the capsid surface.

## Additional Information

**Accession Codes:** Cryo-ET subtomogram averages of P-SSP7 were deposited to EMDataBank with accession numbers EMD-3131 (“parallel”), and EMD-6427 (“leaning”), EMD-1707 (“standing”).

**How to cite this article**: Murata, K. *et al*. Visualizing Adsorption of Cyanophage P-SSP7 onto Marine *Prochlorococcus. Sci. Rep.* **7**, 44176; doi: 10.1038/srep44176 (2017).

**Publisher's note:** Springer Nature remains neutral with regard to jurisdictional claims in published maps and institutional affiliations.

## Supplementary Material

Supplementary Information

Supplementary Movie 1

Supplementary Movie 2

Supplementary Movie 3
