# Supplementary material for: Visualizing Adsorption of Cyanophage P-SSP7 onto Marine Prochlorococcus
Source: Sci Rep. 2017 Mar 10;7:44176. doi: 10.1038/srep44176 (PMC5345008; doi:10.1038/srep44176)
Supplement: Supplementary Information [file srep44176-s1.pdf]

Supplementary Information

Visualizing Adsorption of Cyanophage P-SSP7 onto Marine *Prochlorococcus*

Kazuyoshi Murata[1,3 †], Qinfen Zhang[1,4 †],Jesus Gerardo Galaz-Montoya[1], Caroline Fu[1], Maureen L. Coleman[2,5], Marcia S. Osburne[2,6], Michael F. Schmid[1], Matthew B. Sullivan[2,7,*], Sallie W. Chisholm[2,*], and Wah Chiu[1*]

[1] National Center for Macromolecular Imaging, Verna and Marrs McLean Dept. of Biochemistry & Molecular Biology, Baylor College of Medicine, Houston, TX, 77030

[2] Department of Civil and Environmental Engineering, M.I.T., Cambridge, MA 02139

[3] National Institute for Physiological Sciences, 38 Nishigonaka, Myodaiji, Okazaki, Aichi, 444-8585, Japan

[4] School of Life Sciences, State Key Lab for Biocontrol, Sun Yat-Sen University, Guangzhou, 510275, P. R. China

[5] Current address: Department of the Geophysical Sciences, University of Chicago, Chicago, IL 60637 USA

[6] Current address: Department of Molecular Biology and Microbiology, Tufts University School of Medicine, Boston, MA 02111 USA

[7] Current address: Departments of Microbiology and Civil, Environmental and Geodetic Engineering, The Ohio State University, Columbus, OH 43210 USA

*Correspondence to: wah@bcm.edu, chisholm@mit.edu or mbsulli@gmail.com

[†] Equal contribution.

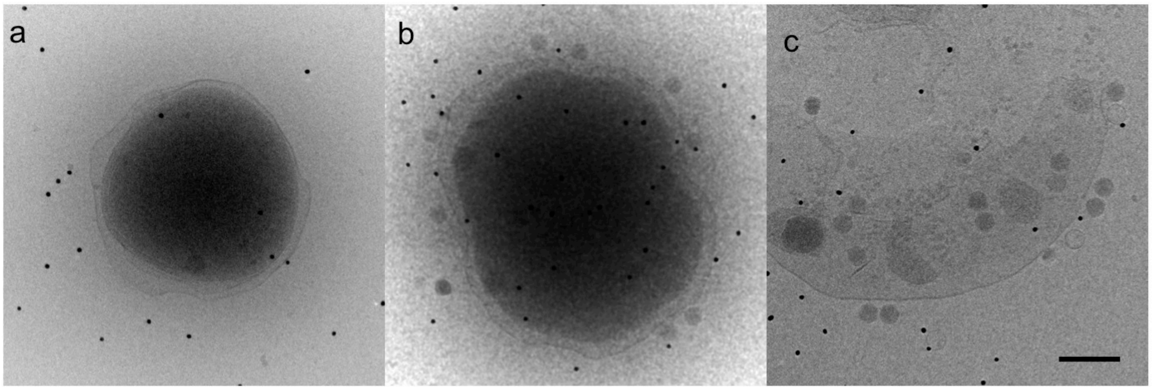

Supplementary Figure 1. Time course analysis of infection of *Prochlorococcus* MED4 by P-SSP7 phages. *Prochlorococcus* MED4 cells were mixed with P-SSP7 phages and observed by cryo-EM at different time points. **(a)** 11 min: MED4 was not approached by P-SSP7 phages. **(b)** 51 min: MED4 started to be adsorbed by P-SSP7phages. **(c)** 86 min: In addition to the infecting phages, post-infecting phages (empty capsid phages) were observed. Further, lytic cells were also observed. The scale bar is 200 nm.

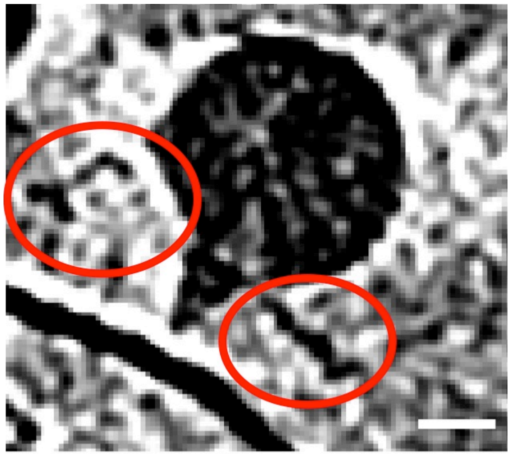

Supplementary Figure 2. A view of representative tail fibers in a single phage. Slice (~10 nm) through a subtomogram of an individual P-SSP7 phage adsorbed onto the MED4 surface, showing strong tail and tail-fiber (red circles) densities. The scale bar is 20 nm.

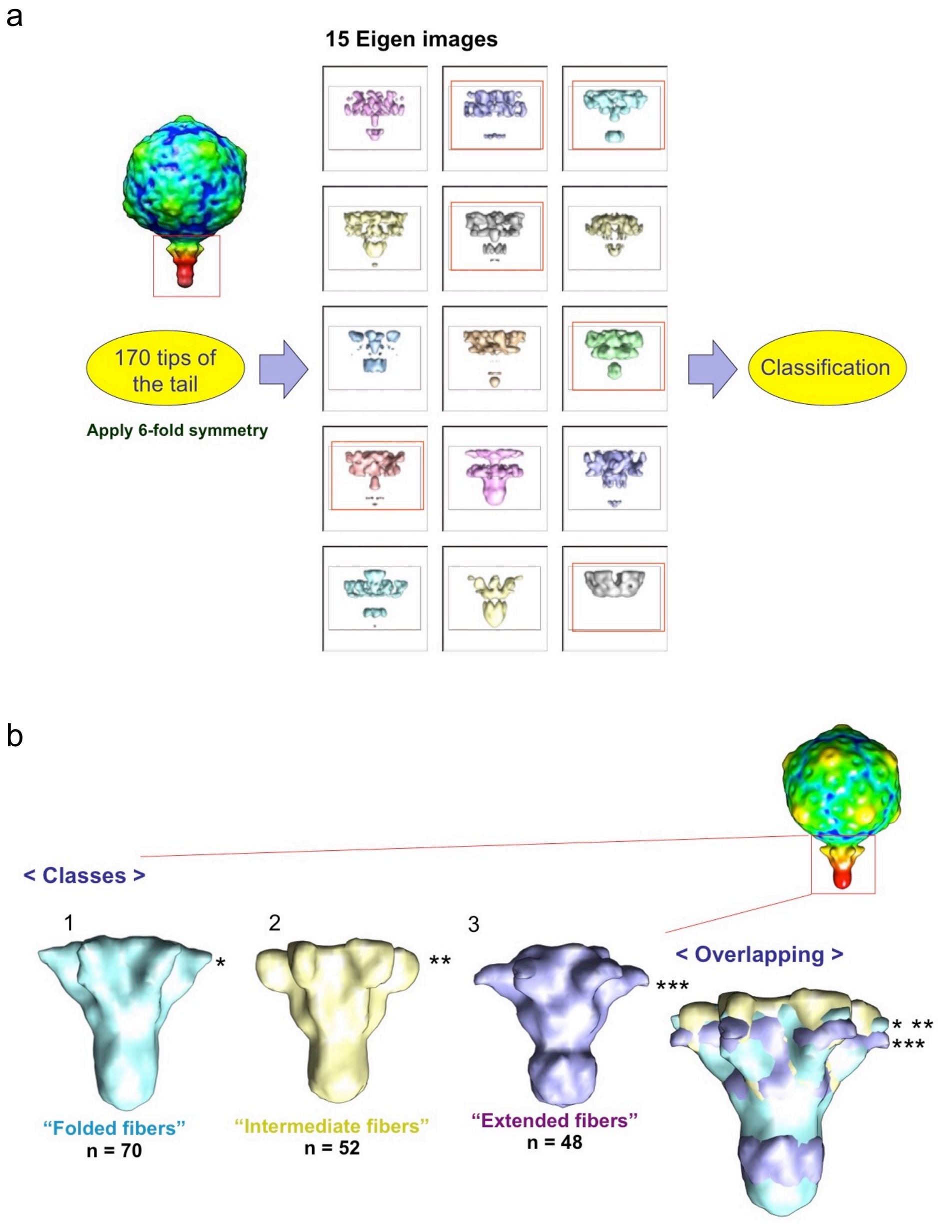

Supplementary Figure 3. 3D classification of tail fiber structures. To investigate conformational changes in tail fibers, the structure of the tail and fibers was subjected to multivariate statistics analysis and classification. **(a)** 170 tail volumes were clipped from P-SSP7 subtomograms, 6-fold symmetrized, and classified according to the characteristic

Eigen images. Initially, 15 Eigen images were generated from the symmetrized tail structures, and six major structures were manually selected as references of 3D classification. **(b)** The great majority of particles preferred three of the six initial references; therefore, we constrained our final classification to these three major classes. Final alignment and averaging yielded three distinct structures, as follows: class1 or “Folded fibers”, class2 or “Intermediate fibers”, and class3 or “Extended fibers”. “Folded fibers” are expected to run along the capsid surface, while “Extended fibers” are expected to extend horizontally. The “Intermediate fibers” are assumed to contain both fiber conformations. While the full length of the fibers is not visible due to limited particle numbers, limited resolution and conformational flexibility, the conformational changes associated with each class are very clear at the base of the fibers, where they are anchored to the tail hub. Furthermore, the tip of the tail hub itself is more spherical in the class average with extended fibers compared to the tail hub in the class average in the folded fiber conformation.

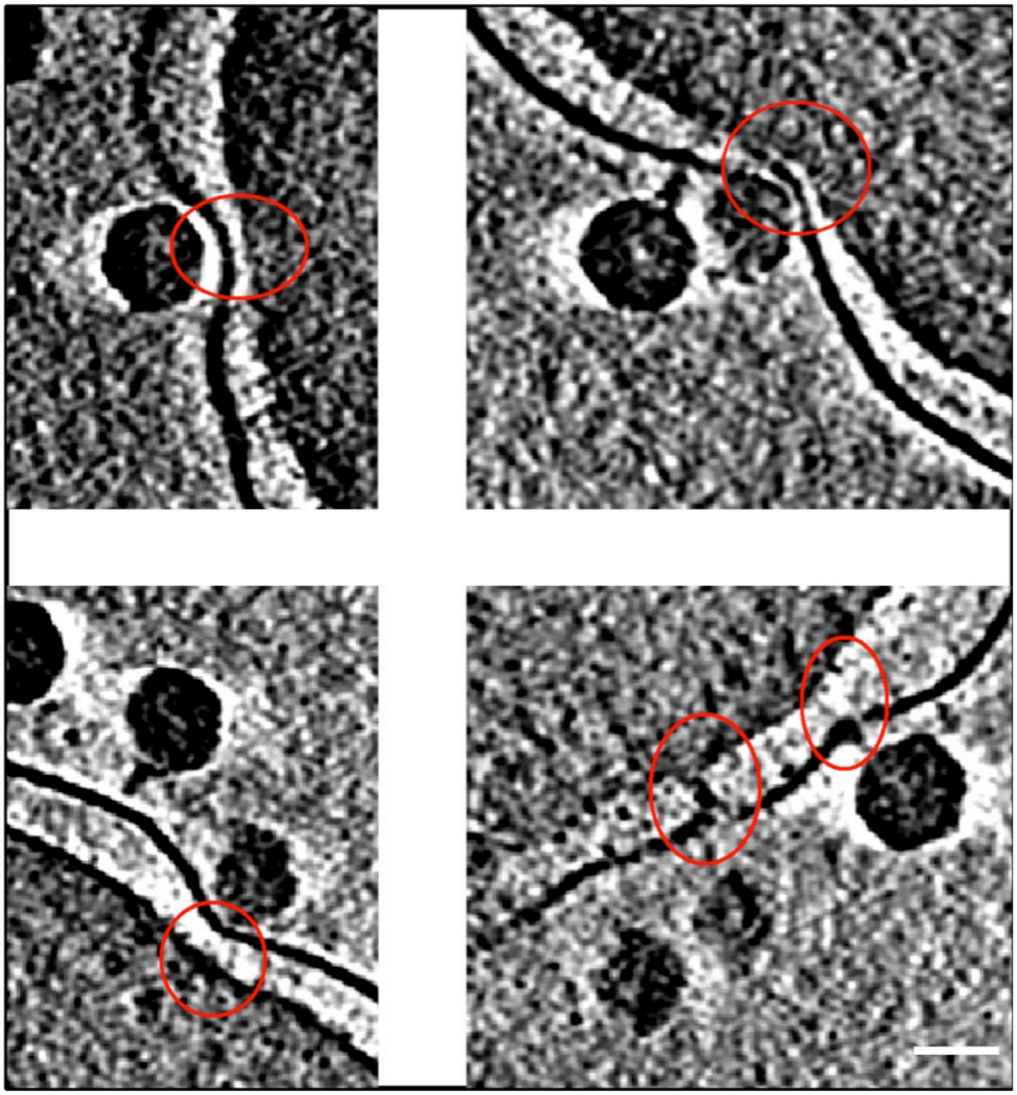

Supplementary Figure 4. Existence of "Adhesion zones"? Examples of regions where the space between the cell wall and membrane is narrowed (red circles). While previous studies with conventional electron microscopy showed abundant "adhesion zones", we only see a few of these per cell. Furthermore, we see many regions where infection-ready viruses are not near adhesion zones (Figure 7). The scale bar is 50 nm

Supplementary Table:

The detailed time information for the sample freezing

No. of grids	Time post infection (minutes)	Time span (minutes)		No. of grids	Time post infection (minutes)	Time span (minutes)		No. of grids	Time post infection (minutes)	Time span (minutes)
1	4			24	42	2		**47**	**96**	**2**
2	6	2		**25**	**44**	**2**		48	98	2
**3**	**11**	**5***		26	46	3		49	100	2
4	14	3		27	49	2		50	101	1
5	17	3		**28**	**51**	**2**		51	118	17*
6	19	2		29	53	2		52	120	2
**7**	**21**	**2**		30	56	3		**53**	**123**	**3**
8	22	1		31	61	5*		54	125	2
**9**	**23**	**1**		32	63	2		55	126	1
10,11	25	2		**33,34**	**67**	**4**		56	128	2
12	27	2		**35**	**68**	**1**				
13	28	1		36,37	69	1		**57**	**381**	**253****
14	29	1		38,39	71	2		58	383	2
15	31	2		40	73	2		59	385	2
16	33	2		**41**	**80**	**7***		60	387	2
**17,18**	**34**	**1**		42	83	3		61	397	10
19,20	36	2		**43**	**86**	**3**		62	399	2
21	38	2		**44**	**89**	**3**		63	401	2
22	39	1		45	92	3		64	403	2
23	40	1		46	94	2				

Note:

Bold: These grids were used for the image analysis of the phage adsorption.

*: The reason for these longer time spans is that the grids in between these time points and the immediately prior time point were obviously bent or broken during freezing procedures. Such unsuitable grids were discarded right away.

**: In order to observe infection at its latest, we incubated the sample for a much longer time and froze it.

Supplementary Movie 1: **Representative tilt series of adsorption of P-SSP7 phage onto *Prochlorococcus* MED4.** Phages mixed with MED4 cells at a multiplicity of infection (MOI) of ~40, were plunge-frozen and collected the tiltseries images with a range of ±62º using a JEM3200FSC electron microscope. The images show adsorption of phages onto a cell at ~86 minutes post-infection.

Supplementary Movie 2: **Z-slices of the 3D-tomogram from the tilt series in Movie S1.** The tiltseries were aligned using gold fiducials, and tomograms were reconstructed using filtered back projection in IMOD software (see Methods).

Supplementary Movie 3: **Annotated figure of the same P-SSP7 adsorbed cell as in Movie S2.** The cell wall and the plasma membrane are red, the thylakoid membranes are dark green, carboxysomes are blue, the polyphosphate body is dark gray, and adsorbed phages on the sides or top of the cell are light green.
